# Strontium ranelate promotes odonto-/osteogenic differentiation/mineralization of dental papillae cells *in vitro* and mineralized tissue formation of the dental pulp *in vivo*

**DOI:** 10.1038/s41598-018-27461-7

**Published:** 2018-06-15

**Authors:** Alamuddin Bakhit, Nobuyuki Kawashima, Kentaro Hashimoto, Sonoko Noda, Keisuke Nara, Masashi Kuramoto, Kento Tazawa, Takashi Okiji

**Affiliations:** Department of Pulp Biology and Endodontics, Division of Oral Health Sciences, Graduate School of Medical and Dental Sciences, Tokyo Medical and Dental University (TMDU), Tokyo, 113-8510 Japan

**Keywords:** Reverse transcription polymerase chain reaction, Calcium signalling, Cell proliferation, Differentiation

## Abstract

This study examined the effects and mechanisms of strontium ranelate (SrRn)—a drug used to treat osteoporosis—on the proliferation and differentiation/mineralization of cloned dental pulp-like cells (mouse dental papillae cells; MDPs). It also determined whether topical application of SrRn to exposed dental pulp tissue promotes the formation of mineralized tissue *in vivo*. The MDPs were cultured with or without SrRn, and cell proliferation, odonto-/osteoblastic gene expression, mineralized nodule formation, and Akt phosphorylation were evaluated. The formation of mineralized tissue in SrRn-treated pulp tissue in rat upper first molars was evaluated histologically. The SrRn up-regulated cell proliferation and expression of *Alp (alkaline phosphatase)*, *Bsp (bone sialoprotein)*, *Dmp (dentin matrix acidic phosphoprotein)-1*, *Dspp (dentin sialophosphoprotein)*, and *Oc (osteocalcin)* in a dose-dependent manner. Mineralized nodule formation was also enhanced by SrRn. NPS-2143, a calcium-sensing receptor (CaSR) antagonist, and siRNA against the CaSR gene blocked SrRn-induced proliferation, odonto-/osteoblastic gene expression, and mineralized nodule formation. SrRn induced Akt phosphorylation, and this was blocked by NPS-2143. Topical application of SrRn to exposed rat molar pulps induced the formation of osteodentin-like mineralized tissue. Our study revealed for the first time that SrRn promotes proliferation and odonto-/osteogenic differentiation/mineralization of MDPs via PI3K/Akt signaling activated by CaSR *in vitro*; mineralized tissue forms from the dental pulp *in vivo*.

## Introduction

Vital pulp therapy has been practiced for over 200 years^1^. This procedure involves the application of a pulp-capping agent onto a remaining thin layer of dentin (indirect pulp capping) or an exposed/excavated pulp tissue (direct capping/pulpotomy)^2,3^. The physical and biological requirements of an ideal direct pulp capping agent include adherence to tooth substrate, maintenance of a sufficient seal, insolubility in tissue fluids, dimensional stability, nontoxicity, lack of carcinogenicity and genotoxicity, radiopacity, and capacity to stimulate hard tissue (tertiary dentin) formation^4–6^.

Calcium hydroxide (CH) and mineral trioxide aggregates (MTA) are the two most popular pulp-capping materials^7^. CH was introduced in the 1930s, and it is the most common agent used in vital pulp therapy^2,8^. CH induces necrosis when applied directly to the pulp tissue due to its high alkalinity. There can be mild inflammation in the subjacent pulp tissue. Next, tissue repair due to inherent reparative capacity of the pulp progresses to form mineralized tissue called a dentin bridge^9^. One drawback of CH is that it is soluble, and the CH-applied area will become “dead space” over time^10^. Furthermore, the first-formed hard tissue of any dentin bridge by CH is irregular with tubular openings or a canalicular lumina containing vessels and cells^11^. These structural defects are termed “tunnel defects”, and they may induce microleakage and subsequent inflammation of the remaining pulp tissue^12^. MTA was originally introduced as a root-end filling material^13^. It has recently been widely investigated as a material for direct pulp capping^14,15^. MTA has a “gentle” hard tissue-inducing capacity^8,16^, and its hard tissue-inducing mechanisms are similar to those of CH^17^. Currently, CH, MTA, and their derivatives are generally used for vital pulp therapy, but more effective pulp capping agents/materials with active dentin-like tissue-inducing capacity might increase the success rate of vital pulp therapy.

Strontium ranelate (SrRn) contains two strontium atoms coupled by ranelic acid. It is used to treat osteoporosis^18^. Previous clinical trials have shown that SrRn significantly improves bone mass/quality and increases bone strength via changes in the bone matrix properties and bone mineral density in osteoporotic patients^18^. SrRn is promising in the treatment of symptomatic osteoarthritis^19^. SrRn has gained attention in osteoporosis therapy because it offers two mechanisms of action, i.e., induction of osteoblastogenesis and suppression of osteoclastogenesis^20,21^. Furthermore, local application of SrRn with a collagen sponge to artificial bone defects formed in rat calvaria promotes the regeneration of bone tissue^22^. These findings indicate the potent mineralized tissue-forming capacity of SrRn and suggest that SrRn can be used on dentin (indirect pulp capping) and dental pulp tissue (direct pulp capping) as a pulp-capping agent with a potent capacity to form mineralized tissue. Thus, the aims of this study were (1) to examine the effects and mechanisms of SrRn on the proliferation and differentiation/mineralization of cloned dental pulp-like cells (mouse dental papilla cells; MDPs); and (2) to determine whether topical application of SrRn to exposed dental pulp tissue promotes mineralized tissue formation *in vivo*.

## Materials and Methods

### Cell culture and chemicals

The MDPs were derived from the incisor apical buds of ICR mice and were immortalized by transfection of human papillomavirus type 16 E6 gene missing the PDZ domain binding motif  ^23,24^. They were cultured in alpha-modified minimum essential medium (α-MEM, Wako Pure Chemical Industries, Osaka, Japan) with 10% fetal bovine serum (FBS, HyClone/GE Healthcare, UT, USA) and an antibiotic and anti-fungal solution (penicillin-streptomycin-amphotericin B suspension; Wako Pure Chemical Industries) at 37 °C/5% CO_2_/100% humidity. SrRn (LKT Laboratories, St Paul, MN, USA) and calcium chloride (CaCl_2_; Wako Pure Chemical Industries) were dissolved in α-MEM directly and sterilized with a syringe filter (Millex-HA filter, Merck Millipore, Darmstadt, Germany). NPS-2143 hydrochloride (dissolved in dimethylsulfoxide, 1.0 µM; Cayman Chemicals, Ann Arbor, MI, USA) and LY294002 (dissolved in dimethylsulfoxide, 1.0 µM; Cayman Chemicals) were used as selective and potent calcium-sensing receptor (CaSR) antagonists^25^ along with a potent pan-PI3K/Akt inhibitor^26^, respectively.

### Proliferation of MDPs

MDPs (5 × 10^3^ cells/well) were seeded in 96-well plates. After 24 h of culturing, the media was changed to contain the test agents. Cell proliferation was measured with the WST-8 assay (CCK-8, Dojindo Molecular Technologies, Kumamoto, Japan) at 48 and 72 h.

### Odonto-/osteoblastic gene expression

MDPs (5 × 10^4^ cells/well) were seeded in 12-well plates. After 24 h of culture, the media was changed to include the test agents, and cells were cultured for 72 h. The total RNA was extracted by QuickGene RNA cultured cell kit S (Wako Pure Chemical Industries). The cDNA was converted from extracted RNA using Primescript (Takara, Shiga, Japan). The qPCR assays were performed with specific primers (Table 1) and GoTaq qPCR Master Mix (Promega, Madison, WI, USA) using a CFX96 Real-Time PCR Detection Systems (Bio-Rad, Hercules, CA, USA). Gyceraldehyde-3-phosphate dehydrogenase (Gapdh) was the internal control.

**Table 1:** Primer sequences.

Genes	Upper Primers	Lower Primers	Gene bank No.	Size
*Gapdh*	5′-TGACGACTTCAACAGCAACTC-3′	5′-ATGTAGGCCAATGAGGTCCAC-3′	BC096590	143
*Alp*	5′-GATTACGCTCACAACAACTACCAG-3	5′-GGAATGTAGTTCTGCTCATGGAC-3′	NM_007431	140
*BSP*	5′-TATGAAGTCTATGACAACGAGAACG-3	5′-AGTAATAATTCTGACCCTCGTAGCC-3′	NM_008318	121
*CaSR*	5′-AATGACACTTTGAACAGACACCAG-3′	5′- ATTTCATCTGGGCTTTCTATTTCTG-3′	NM_013803.3	139
*Dmp-1*	5′-CGTTCTGAGGAAGACAGTGACTC-3′	5′-TTAGTTTCCTACTGTCAGCTTCCAT-3′	NM_016779	130
*Dspp*	5′-AAGGATAGCAGTTCTGACAGCAG-3′	5′-AATCATCACTGGTTGAGTGGTTCCT-3′	NM_010080	136
*Oc*	5′-CATACTGGTCTGATAGCTCGTCAC-3′	5′-AGGGCAATAAGGTAGTGAACAGAC-3′	NM_007541	129

### Mineralized nodule formation

MDPs (1 × 10^4^ cells/well) were seeded in 48-well plates and cultured in Opti-MEM (Thermo Fisher Scientific, Waltham, MA, USA) containing 10% FBS. After 24 h of culturing, the media was changed to an osteogenic medium containing L-ascorbic acid (0.2 mM; Wako Pure Chemical Industries) and β-glycerophosphate (5.0 mM; Sigma Aldrich, St. Louis, MO, USA) with or without test agents. Mineralized nodules were stained with alizarin red S (Wako Pure Chemical Industries) after 7 d of culture. The density of the mineralized nodules was measured by ImageJ software v2 (https://imagej.net/ImageJ2).

### Western blotting

MDPs (2 × 10^5^ cells/well) were seeded in 24-well plates. Test agents were added to the media after 24 h of culture. Cells were lysed with a RIPA buffer (20 mM Tris/HCl pH 7.4, 150 mM NaCl, 10 mM MgCl_2_, 5 mM EDTA, 1% NP-40, 5% glycerol) containing cOmplete Ultra (Merck Millipore) and PhosSTOP EASY (Merck Millipore). Cell lysates were applied to SDS-PAGE (10%), and the gel was transferred to a PVDF membrane (Immobilon-P, Merck Millipore) using a semi-dry transfer system (0.15 mA, 1 h; WSE-4040, ATTO, Tokyo, Japan). Transferred membranes were incubated with anti-Akt (1:1000; polyclonal, GTX121936, GeneTex, Los Angeles, CA, USA) and anti-p-Akt (1:500; polyclonal, GTX121936, GeneTex) antibodies overnight at 4 °C followed by horseradish peroxidase (HRP)-conjugated anti-rabbit IgG (1:1000; Jackson Immuno Research Labs, West Grove, PA, USA) for 30 min at room temperature. Gapdh was used as a loading control (HRP-conjugated mouse anti-GAPDH, 1:1000; Clone: 3H12, Medical & Biological Laboratories Co., LTD., Tokyo, Japan). Chemiluminescent detection used an HRP substrate (Immobilon, Merck Millipore) and a digital autoradiograph imaging system (LAS-3000, Fujifilm, Tokyo, Japan). The pixel density was measured with ImageJ software v2.

### siRNA transfection of the CaSR in MDPs

MDPs (1 × 10^5^ cells/well) were seeded in 12-well plate, and CaSR siRNA (#s201124; Thermo Fisher Scientific) or negative control (siNC, Ambion Silencer Negative Control #1 siRNA, Thermo Fisher Scientific) were transfected with Lipofectamine RNAiMAX transfection reagent (Thermo Fisher Scientific). After 12 h of culture, the medium was changed to include the test agents, and the cells were cultured for 72 h.

### *In vivo* study

All animal experiments were approved by the Animal Care and Use Committee of TMDU, all surgical methods were performed in accordance with relevant ethical guidelines and regulations (#A2017-155A). Wistar rats (n = 12, male, 5-wk-old; Clea Japan, Tokyo, Japan) were given *ad libitum* access to food and water prior to the experiment. The rats were anesthetized with an intraperitoneal injection of ketamine (90 mg/kg) and xylazine (10 mg/kg). The cavity preparation and pulp exposure were performed in the upper first molars of both sides with #1/2 round bars using a dental handpiece motor under a stereoscopic microscope (Dental Microscope Z; Mani, Tochigi, Japan). Bleeding in the cavities following the pulp exposure was removed with sterile cotton pellets. The SrRn (mixed with sterile water at 2 mg/µl), mineral trioxide aggregate (ProRoot MTA, Dentsply Sirona, Ballaigues, Switzerland; mixed according to the manufacturer’s instructions), or CaCl_2_ (mixed with sterile water at 2 mg/µl) was dressed over the exposed pulp (n = 4 in each group). No application of SrRn, MTA, and CaCl_2_ was used as a control. The samples applied to each cavity were randomly chosen from SrRn, MTA, CaCl_2_, and no application; there was no rat in which the same samples were applied contra-laterally. The cavities were sealed with glass ionomer cement (Ionosit-Baseliner, DMG, Hamburg, Germany). The rats were sacrificed by CO_2_ euthanasia after 3 weeks. The upper jaws were dissected from the maxilla and fixed with 4% paraformaldehyde/PBS for 24 hours at 4 °C. Samples were then demineralized using 17% EDTA (Dojindo Molecular Technologies) for 3 weeks. After demineralization, the glass ionomer cement was removed, and the samples were embedded in paraffin. Hematoxylin and eosin staining was performed on 5 µm-thick sections, and the stained sections were observed under a light microscope (Axio Vert.A1, Carl Zeiss, Oberkochen, Germany).

### Statistical Analysis

All *in vitro* experiments were carried out in triplicates. The data were submitted to one-way ANOVA followed by Tukey’s test. The level of significance was established at ^*^P < 0.05 or ^**^p < 0.001 using Prism software v7 (GraphPad, San Diego, CA, USA).

## Results

### SrRn promoted cell proliferation and differentiation/mineralization of MDPs

**Figure 1:**
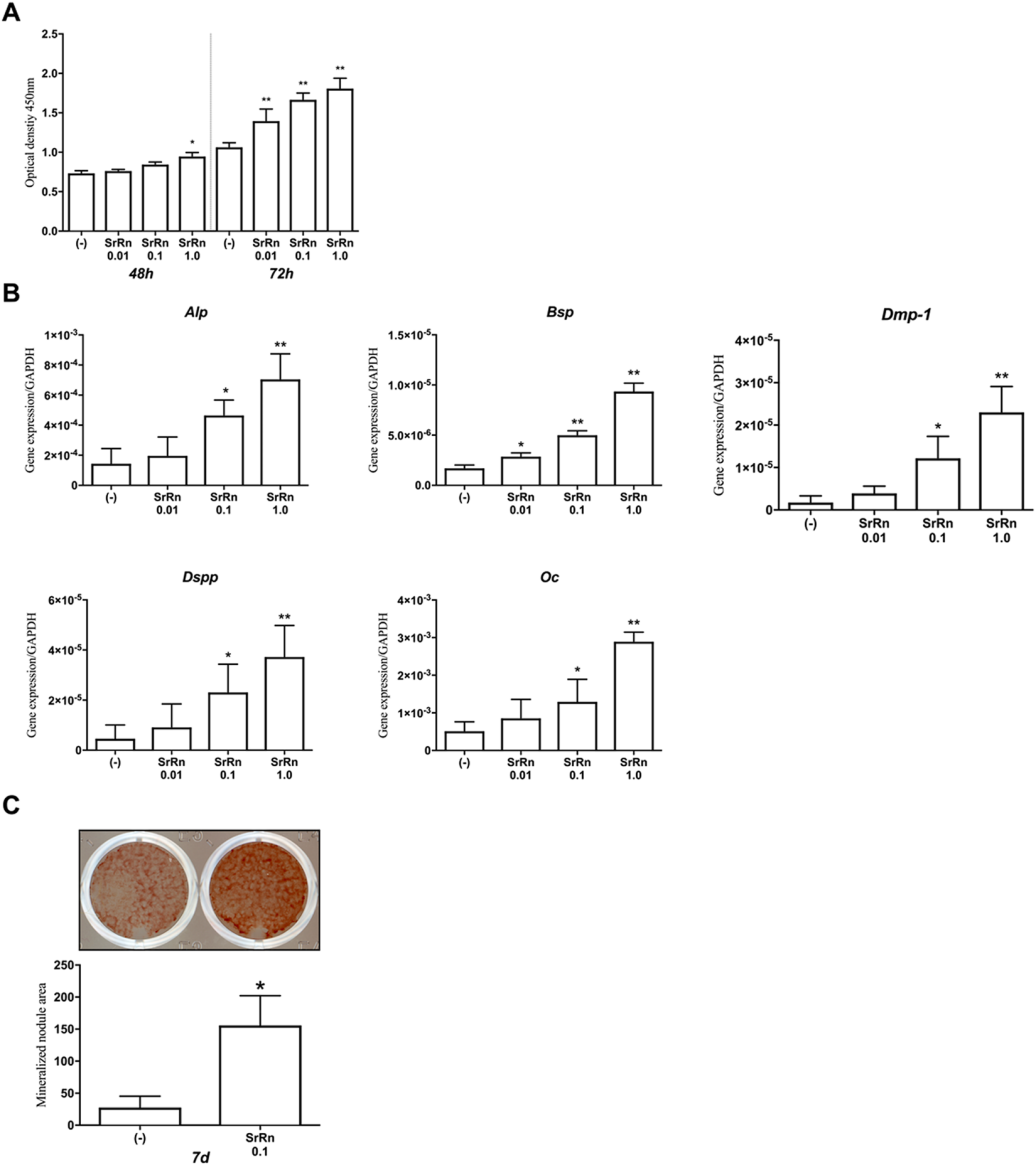
The effect of SrRn on proliferation, odonto-/osteogenic differentiation, and mineralization of MDPs. (**A**) Proliferation of MDPs was increased by SrRn at 48 and 72 h. (**B**) mRNA expression of *Alp*, *Bsp*, *Dmp-1*, *Dspp*, and *Oc* in MDPs was up-regulated by SrRn. (**C**) Mineralized nodule formation increased in MDPs cultured in the osteogenic medium with SrRn (0.1 mM) for 7 d. ^*^P < 0.05 or ^**^p < 0.001 compared to control.

First, we examined the effect of SrRn on the growth, odonto-/osteoblastic gene expression and mineralized nodule formation of MDPs. SrRn significantly increased the proliferation of MDPs at 48 and 72 h in a dose-dependent manner (Fig. 1A). Expression of *Alp (alkaline phosphatase)*, *Bsp (bone sialoprotein)*, *Dmp (dentin matrix acidic phosphoprotein)-1*, *Dspp (dentin sialophosphoprotein)*, and *Oc* *(osteocalcin)* was also upregulated by SrRn in a dose-dependent manner (Fig. 1B). Osteogenic medium containing SrRn (0.1 mM) induced mineralized nodule formation (Fig. 1C). The CaCl_2_ failed to induce cell proliferation and mineralized nodule formation (see Supplemental Fig. 1).

### CaSR is involved in the up-regulation of cell proliferation and differentiation/mineralization of MDPs induced by SrRn

**Figure 2:**
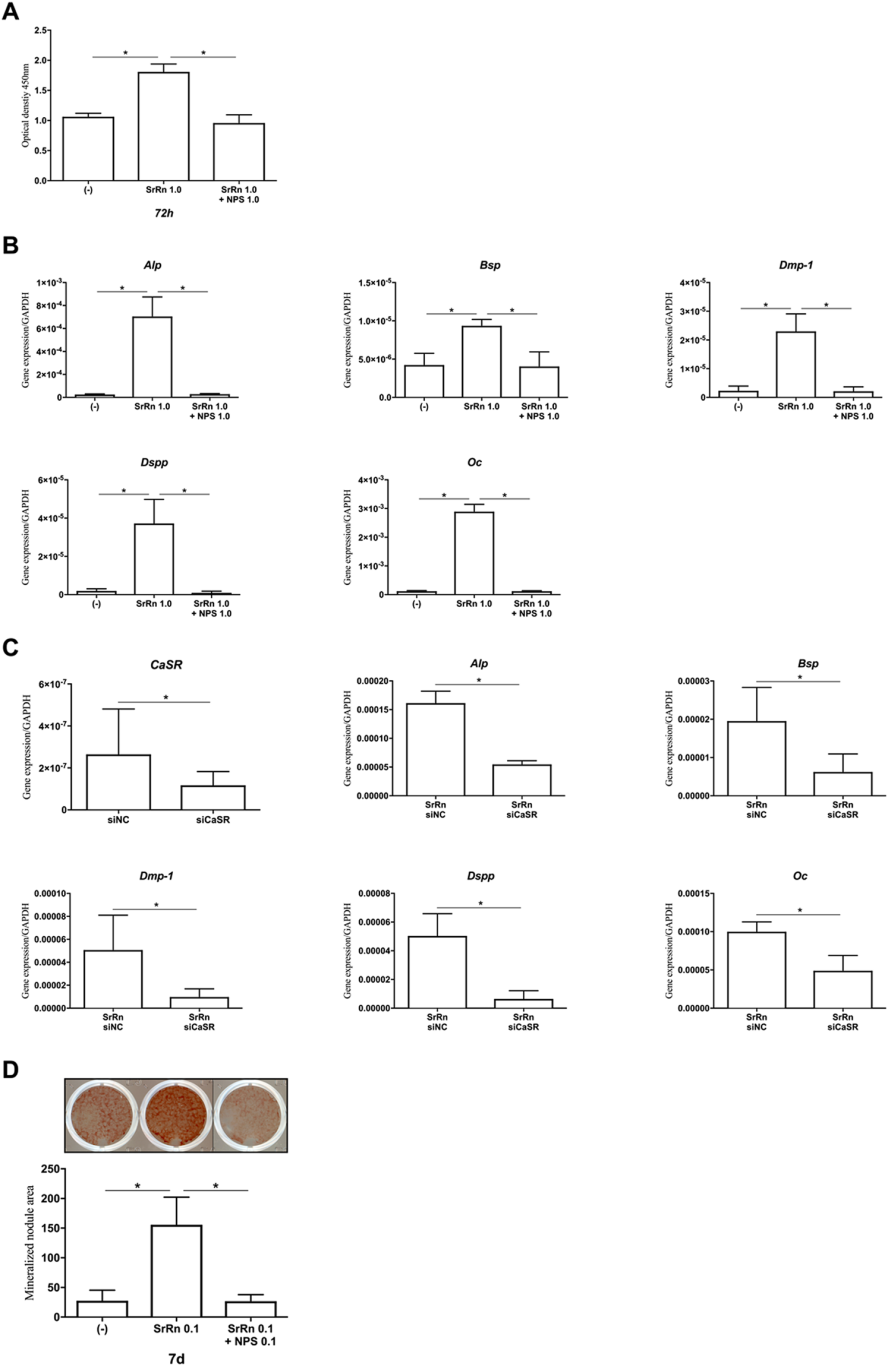
The effect of CaSR inhibition on enhanced proliferation, odonto-/osteoblastic gene expression, and mineralized nodule formation of MDPs induced by SrRn. (**A**) Proliferation of MDPs enhanced by SrRn (1.0 mM) was blocked by NPS-2143 (1.0 µM). (**B**) mRNA expression of *Alp*, *Bsp*, *Dmp-1*, *Dspp*, and *Oc* in MDPs by SrRn (1.0 mM) was blocked by NPS-2143 (1.0 µM) at 72 h. (**C**) siRNA CaSR down-regulated the mRNA expression of CaSR in MDPs. Expression of *Alp*, *Bsp*, *Dmp-1*, *Dspp*, and *Oc* promoted by SrRn in MDPs was blocked by siCaSR. (**D**) Mineralized nodule formation promoted by SrRn in MDPs was down-regulated by NPS-2143. NPS: NPS-2143, siNC: negative control of siRNA, and siCaSR: siRNA of CaSR. ^*^P < 0.05 compared with each other.

Next, we investigated the possibility that CaSR acts as a targets of SrRn in MDPs, because Sr^2+^ is known to activate CaSR^27,28^, which is involved in the control of many important cellular functions such as proliferation and differentiation^29^. The promoted cell proliferation and expression of *Alp*, *Bsp*, *Dmp-1*, *Dspp*, and *Oc* induced by SrRn on MDPs were disrupted by NPS-2143—a selective and potent CaSR antagonist (Fig. 2A,B). The CaSR siRNA down-regulated the mRNA expression of *CaSR* in MDPs and also suppressed the expression of *Alp*, *Bsp*, *Dmp-1*, *Dspp*, and *Oc* induced by SrRn in MDPs (Fig. 2C). Mineralized nodule formation promoted by SrRn in MDPs was blocked by NPS-2143 (Fig. 2D).

### PI3K/AKT signaling was activated by SrRn via CaSR

**Figure 3:**
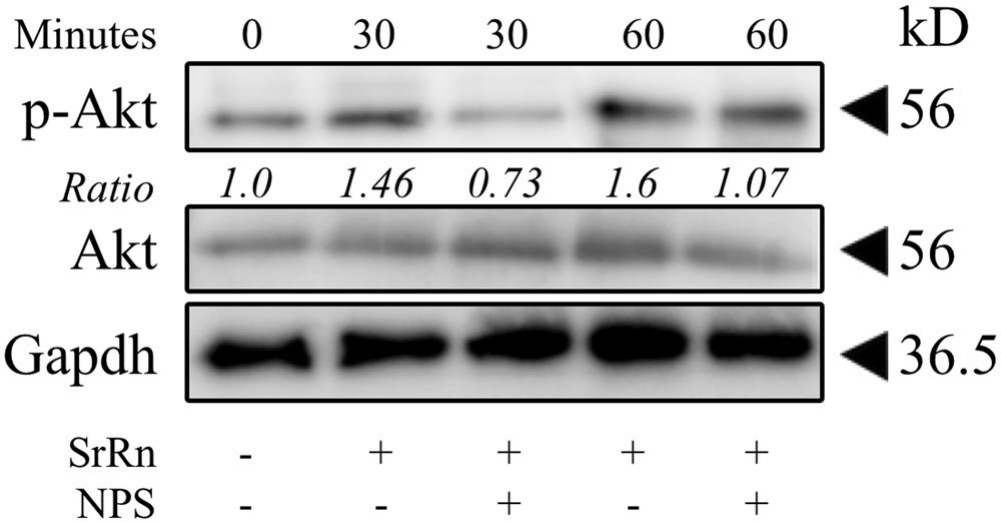
The effect of SrRn on Akt signaling in MDPs. The expression of p-Akt in MDPs was promoted by SrRn, which was down-regulated by NPS-2143. Bands of p-Akt and Akt were quantitated using densitometry, and their ratios were calculated. The experiment was performed in triplicates, and typical results are shown. NPS: NPS-2143.

**Figure 4:**
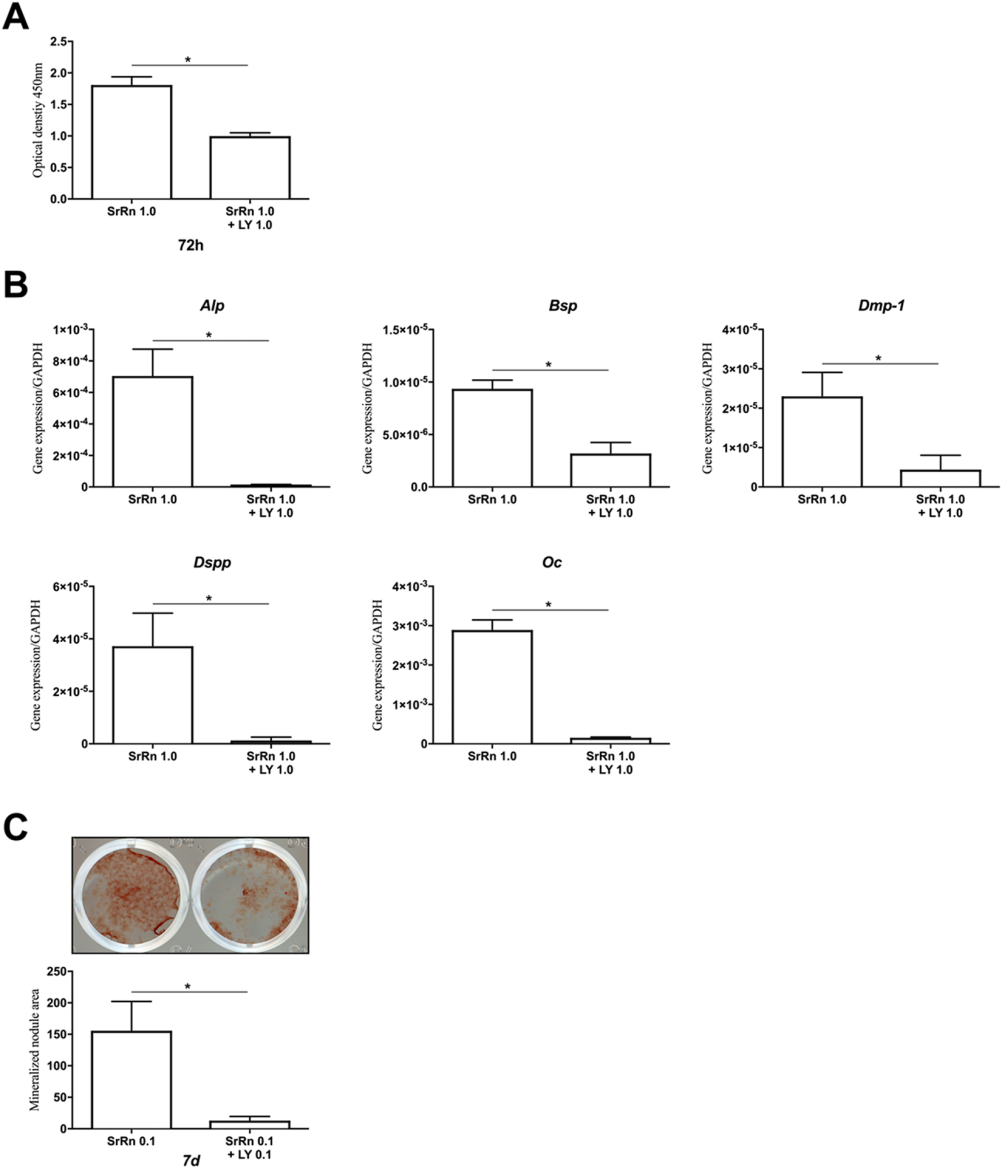
The effect of a PI3K/AKT inhibitor on SrRn-induced proliferation, differentiation and mineralized nodule formation in MDPs. (**A**) Proliferation of MDPs enhanced by SrRn is down-regulated by LY294002. (**B**) LY294002 down-regulated the expression of *Alp*, *Bsp*, *Dmp-1*, *Dspp*, and *Oc* promoted by SrRn in MDPs. (**C**) Mineralized nodule formation promoted by SrRn in MDPs was down-regulated by LY294002. LY: LY294002, ^*^P < 0.05 compared with each other.

We further sought to demonstrate intracellular pathways linked to CaSR in SrRn-stimulated MDPs and investigated PI3K/AKT signaling, which is known as a major signaling pathway of CaSR in osteoblasts^27,30^. SrRn (0.1 mM) induced upregulation of Akt phosphorylation in MDPs at 30 and 60 minutes, which was blocked by NPS-2143 (Fig. 3). LY294002-a potent pan-PI3K/Akt inhibitor, downregulated cell proliferation (Fig. 4A), expression of *Alp*, *Bsp*, *Dmp-1*, *Dspp*, and *Oc* (Fig. 4B) as well as mineralized nodule formation (Fig. 4C) that was promoted by SrRn.

### SrRn promoted mineralization of the exposed rat molar pulp *in vivo*

**Figure 5:**
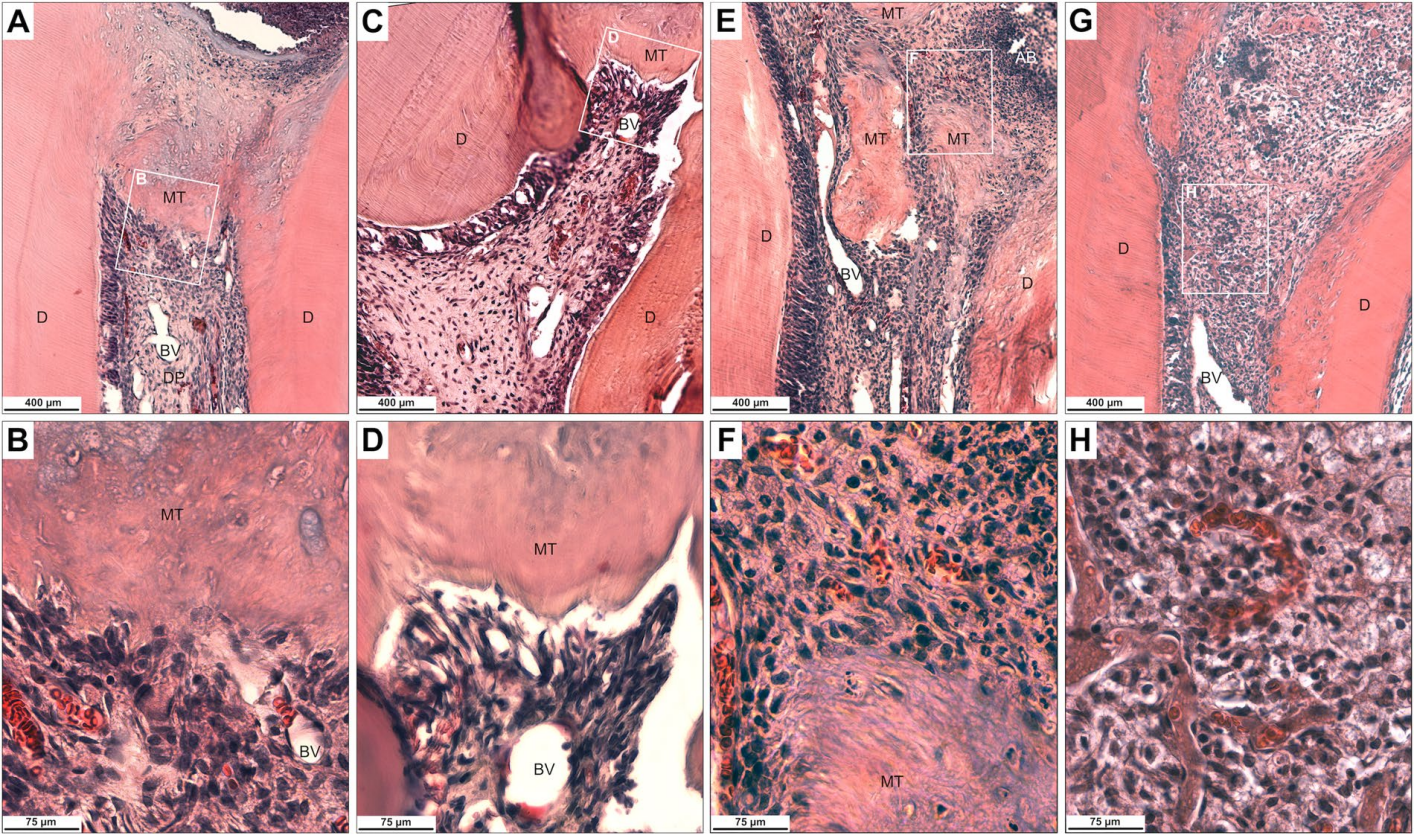
Mineralized tissue formation by topical application of SrRn to the exposed pulp tissue in rat upper first molars. H&E staining in (**A**) shows that mineralized tissue was newly formed in the coronal part of the pulp tissue after the application of SrRn. (**B**) A high-magnification view of the boxed area indicated in A. (**C**) New mineralized tissue was also formed in the coronal part of the pulp tissue after application of MTA. (**D**) A high-power view of the boxed area indicated in C. (**E**) Islets of mineralized tissues—but not continuous mineralized tissue—was formed in the pulp tissue after application of CaCl_2._ An abscess is formed in the exposed area of pulp tissue. (**F**) A high-power view of the boxed area indicated in E. (**G**) No mineralized tissue was formed in non-application control. (**H**) A high-power view of the boxed area indicated in G. AB; Abscess, BV; Blood vessel, D; Dentin, DP; Dentin pulp, MT; newly formed mineralized tissue.

Finally, the impact of topical application of SrRn on exposed dental pulp tissue including the formation of mineralized tissue was examined *in vivo*. The application of SrRn to the exposed pulp tissue of rat upper first molars induced mineralized tissue formation in the pulp tissue of all specimens (Fig. 5A,B). The newly formed mineralized tissue exhibited an atubular, osteodentin-like structure with sparse or no cellular inclusions in the inner (pulp side) portion. This new tissue completely separated the pulp tissue from the pulp chamber exposed to the oral cavity. The pulp tissue appeared mostly normal with a few inflammatory cells.

MTA also induced mineralized tissue formation in the exposed pulp tissue in all specimens; the mineralized tissue had irregularly aligned tubules and fewer cellular inclusions compared to the SrRn-applied specimens. The underlying pulp tissue appeared essentially normal (Fig. 5C,D). In contrast, application of CaCl_2_ failed to induce continuous mineralized tissue formation; some islets of mineralized tissues were formed in the dental pulp tissue. Abscess formation was detected in the exposed area of the pulp tissue (Fig. 5E,F). Some of the CaCl_2_-applied samples showed deposition of mineralized tissues on the surface of the root canal wall dentin. Controls without treatment (Fig. 5G,H) failed to form mineralized tissue in the exposed pulp tissue. Four specimens were examined, and the results were reproducible.

## Discussion

In this study, SrRn up-regulated the proliferation of MDPs (Fig. 1A), which possess dental pulp cell properties^23,24^. SrRn also increased the production of human dental pulp cells (see Supplemental Fig. 2). This is consistent with prior findings showing that SrRn up-regulates the proliferation of MC3T3-E1 cells (a mouse calvaria derived osteoblastic cell line^28,31^) and a primary osteoblast cell line^32^. However, the opposite results have also been reported for MC3T3-E1 cells^33^. The reason(s) for such discrepancy remains unclear, but there is a large variation among the sub-clones of MC3T3-E1^34^, and differences in the properties of different MC3T3-E1 sub-clones may result in different results during cell proliferation. The SrRn also promoted the odonto-/osteoblastic gene expression in a dose-dependent manner (Fig. 1B) and induced mineralization in MDPs (Fig. 1C). Osteoblastic differentiation by SrRn is reported in primary murine bone cells^21^, murine marrow stromal cells^35^, and MC3T3-E1 cells^36^. SrRn also induces commitment of osteoblastic differentiation from mesenchymal stem cells^37^.

One of the targets of Sr^2+^ is CaSR—a Class C G-protein coupled receptor^29^ expressed on various cells including osteoblasts and their precursors^38^. Osteoblasts from the conditional knockouts of CaSR exhibit delayed differentiation, reduced mineralization capacity, and altered expression of regulators of mineralization^39^. This suggests that CaSR signaling in osteoblasts is essential for their differentiation and mineralization. The enhanced cell proliferation, odonto-/osteogenic differentiation and mineralization induced by SrRn in MDPs were blocked by NPS-2143 (a potent CaSR antagonist) and CaSR siRNA (Fig. 2A–D). The NPS-2143 also inhibited the SrRn-induced proliferation of human dental pulp cells (see Supplemental Fig. 2). Furthermore, SrRn promoted CaSR expression in MDPs (see Supplemental Fig. 3). These results indicate that SrRn may promote the proliferation and differentiation/mineralization of MDPs via CaSR signaling.

**Figure 6:**
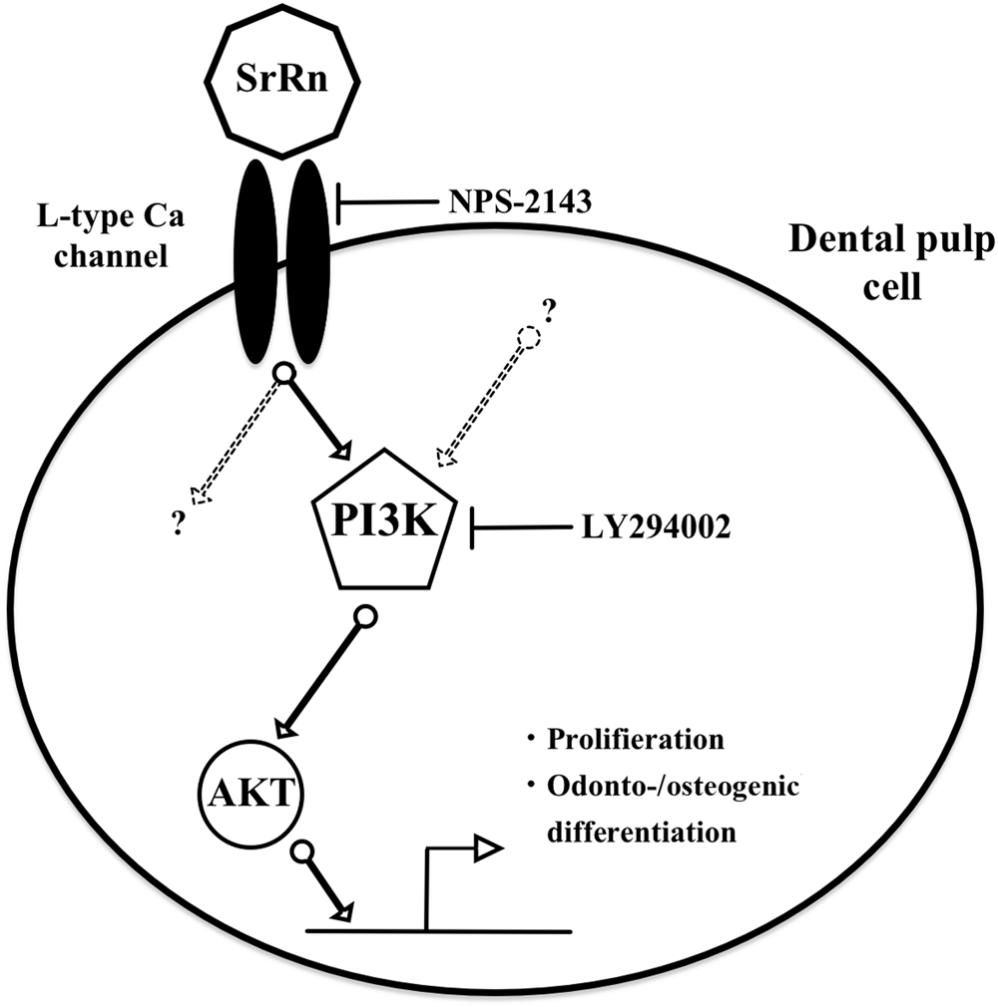
A schematic illustration of the proposed effects of SrRn on MDPs. SrRn releases Sr^2+^ ions entering the cytoplasm through the CaSR, and then activates the PI3K/AKT signaling pathway to promote proliferation and odonto-/osteogenic differentiation of MDPs.

Downstream of CaSR, the phospholipase C (PLC)/inositol-1,4,5-trisphosphate (InsP3) signaling pathway^40^ is widely studied, but Sr^2+^ is less potent than Ca^2+^ in stimulating inositol phosphate accumulation^41^. CaSR has also been linked to several signaling pathways such as the extracellular-signal-regulated kinases (ERKs) 1/2^42,43^ and Jun amino-terminal kinase (JNK)^44^. The SrRn induced the phosphorylation of the Akt in MDPs (Fig. 3), and this was abolished by NPS-2143 (Fig. 3). The PI3K/Akt inhibitor LY294002 disrupted the enhanced proliferation, differentiation, and mineralization by SrRn (Fig. 4) suggesting that the PI3K/Akt pathway is a major signaling cascades of CaSR in MDPs (Fig. 6).

Involvement of the PI3K/Akt pathway is also reported in SrRn-induced osteogenic differentiation of human osteoblasts^30^. The PI3K/Akt signaling stimulates canonical Wnt signaling^37^, and this may be involved in promoted proliferation, differentiation, and mineralization of dental pulp cells due to SrRn. We previously reported that Wnt signaling upregulates odontoblast marker expression in MDPs^45^, and Wnt signaling activated by SrRn via CaSR/PI3K/Akt signaling may induce odonto-/osteoblastic gene expressions of MDPs. The PI3K/Akt signaling activates the production of BMP2^46^, which may also be involved in the differentiation and mineralization of MDPs. Ca^2+^ is an agonist of the CaSR with a higher affinity than Sr^2^^+^^41^; CaCl_2_ (2 mM) promotes the proliferation of MC3T3-E1 cells^28^. However, CaCl_2_ (1 mM) did not enhance the proliferation and differentiation/mineralization of MDPs in our experiment (see Supplemental Fig. 1) suggesting that 1 mM of Ca^2+^ concentration may be too low to induce proliferation and differentiation/mineralization of MDPs.

*In vivo* application of SrRn to rat upper first molars induced tissue mineralization along the exposed pulp tissue (Fig. 5A,B). Mineralized tissues formed in CH-treated pulp tissue have been reported to be irregular with tubular openings or canalicular lumina containing vessels and cells^11^. The mineralized tissue induced by SrRn in this study had an atubular, osteodentin-like structure with sparse or no cellular inclusions in its mineralized structure indicating that it might have sufficient capacity to protect the underlying pulp tissue from the oral environment. The MTA induced dentin-like mineralized tissue (Fig. 5C,D) as previously reported^14,15^, and it offers slow-release of Ca^2+^^47^. In contrast, CaCl_2_ failed to induce homogeneous mineralized tissue did form islets of mineralized bodies in the exposed pulp tissue (Fig. 5E,F). Ca^2+^ is reported to have mineralized tissue formation capacity^28,47^, which agrees with these findings. However, CaCl_2_ did not induce a continuous mineralized barrier to separate the intact pulp tissue from the oral cavity suggesting that an application method resulting in continuous and slow release of Ca^2+^ might be essential to the formation of a mineralized dentin-like barrier.

Systemic side effects should always be considered in developing new treatment agents. Lithium chloride (LiCl_2_)—a GSK3b inhibitor and potent activator of Wnt canonical signaling—has recently been reported to induce mineralized tissue formation following direct application to the rat pulp tissue^48^; however, a high intake of LiCl_2_ can cause a risk of confusion and speech impairment including a risk of death at 20 mg/L of LiCl_2_^49^.

The systemic side effects of SrRn are also of note and include cerebrovascular disease, peripheral vascular disease, and prior myocardial infarction. These were reported in patients given a 2,000 mg daily systemic dose treatment of SrRn for a year^50^. However, it is reasonable to suppose that topical application during vital pulp therapy may pose a much lower risk of side effects compared to systemic application^32,37,51,52^; *in vivo* studies using rat models reported that systemic use of SrRn (625 g/kg) is well tolerated and safe with no adverse side effects^53,54^. However, it is necessary to evaluate the systemic side effects induced by the local application of SrRn before its clinical application.

In conclusion, we revealed for the first time that SrRn increases the proliferation, odonto-/osteoblastic gene expression, and mineralized nodule formation of dental pulp-like cells. This may be partially mediated by CaSR/PI3K/Akt signaling (Fig. 6). Our *in vivo* study revealed that topical application of SrRn induced a continuous barrier of osteodentin-like mineralized tissue on the rat exposed pulp tissue highlighting the potential utility of SrRn as a new pulp-capping material.

## Electronic supplementary material

Supplementary figures 1-3
