# Author Correction: Strontium ranelate promotes odonto-/osteogenic differentiation/mineralization of dental papillae cells *in vitro* and mineralized tissue formation of the dental pulp *in vivo*

**DOI:** 10.1038/s41598-020-65509-9

**Published:** 2020-05-15

**Authors:** Alamuddin Bakhit, Nobuyuki Kawashima, Kentaro Hashimoto, Sonoko Noda, Keisuke Nara, Masashi Kuramoto, Kento Tazawa, Takashi Okiji

**Affiliations:** Department of Pulp Biology and Endodontics, Division of Oral Health Sciences, Graduate School of Medical and Dental Sciences, Tokyo Medical and Dental University (TMDU), Tokyo, 113-8510 Japan

Correction to: *Scientific Reports* 10.1038/s41598-018-27461-7, published online 15 June 2018

This Article contains errors.

In Table 1, the fourth column “Gene bank No.”

should read:

“Accession No.”

Additionally, row Gapdh is incorrect,

“5′-TGACGACTTCAACAGCAACTC-3”

should read:

“5′-TGCGACTTCAACAGCAACTC-3”

“5′-ATGTAGGCCAATGAGGTCCAC-3′”

should read:

“5′-ATGTAGGCCATGAGGTCCAC-3′”

“BC096590”

should read:

“NM_008084”

The Accession No. for *CaSR*,

“NM_013803.3”

should read:

“NM_013803”

Furthermore, in the Discussion section,

“However, it is reasonable to suppose that topical application during vital pulp therapy may pose a much lower risk of side effects compared to systemic application^32,37,51,52^; *in vivo* studies using rat models reported that systemic use of SrRn (625 g/kg) is well tolerated and safe with no adverse side effects^53,54^.”

should read:

“However, it is reasonable to suppose that topical application during vital pulp therapy may pose a much lower risk of side effects compared to systemic application^32,37,51,52^; *in vivo* studies using rat models reported that systemic use of SrRn (625 mg/kg) is well tolerated and safe with no adverse side effects^53,54^.”

