# Supplementary material for: Strontium ranelate promotes odonto-/osteogenic differentiation/mineralization of dental papillae cells in vitro and mineralized tissue formation of the dental pulp in vivo
Source: Sci Rep. 2018 Jun 15;8:9224. doi: 10.1038/s41598-018-27461-7 (PMC6003917; doi:10.1038/s41598-018-27461-7)
Supplement: Supplementary file 1 — Supplementary figures 1-3 [file 41598_2018_27461_MOESM1_ESM.pdf]

Strontium ranelate promotes odonto-/osteogenic differentiation/mineralization of dental papillae cells *in vitro* and mineralized tissue formation of the dental pulp *in vivo*

Alamuddin Bakhit, Nobuyuki Kawashima, Kentaro Hashimoto, Sonoko Noda, Keisuke Nara, Masashi Kuramoto, Kento Tazawa, and Takashi Okiji

A

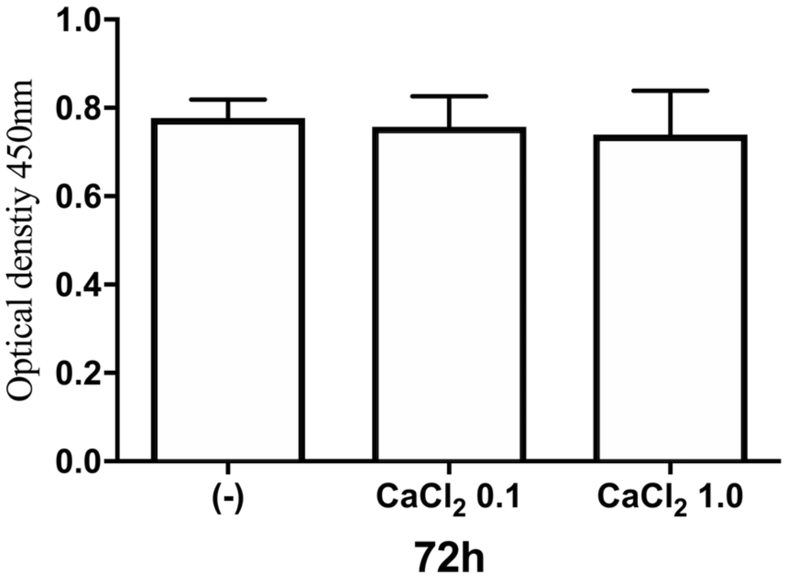
1.0
0.8
0.6
0.4
0.2
0.0
Optical denstiy 450nm
(-)
$CaCl_2$ 0.1
$CaCl_2$ 1.0
72h

B

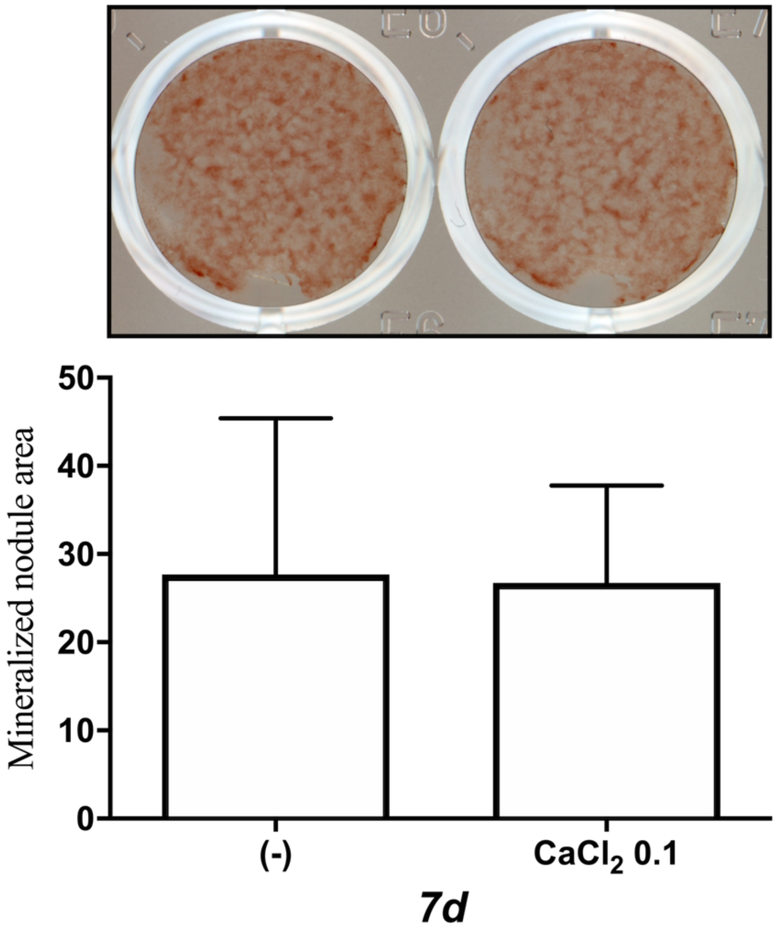
50
40
30
20
10
0
Mineralized nodule area
(-)
$CaCl_2$ 0.1
7d

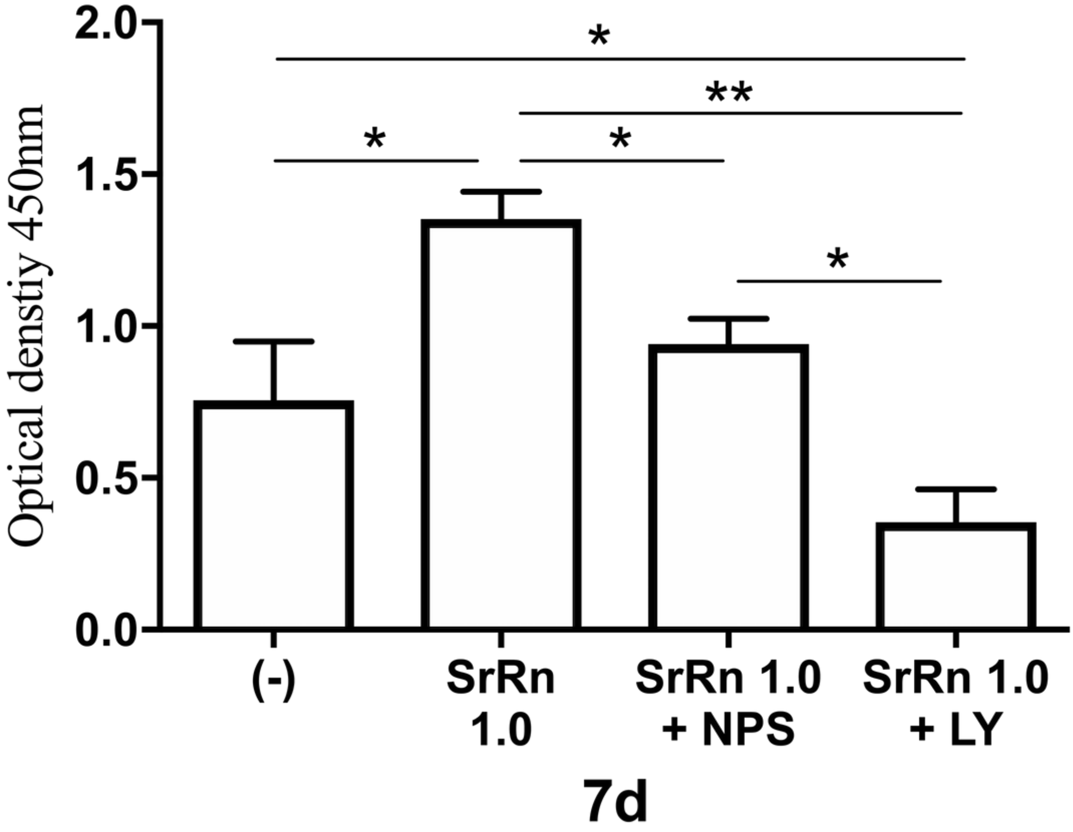
2.0
1.5
1.0
0.5
0.0
Optical denstiy 450nm
*
**
*
*
*
(-)
SrRn 1.0
SrRn 1.0 + NPS
SrRn 1.0 + LY
7d

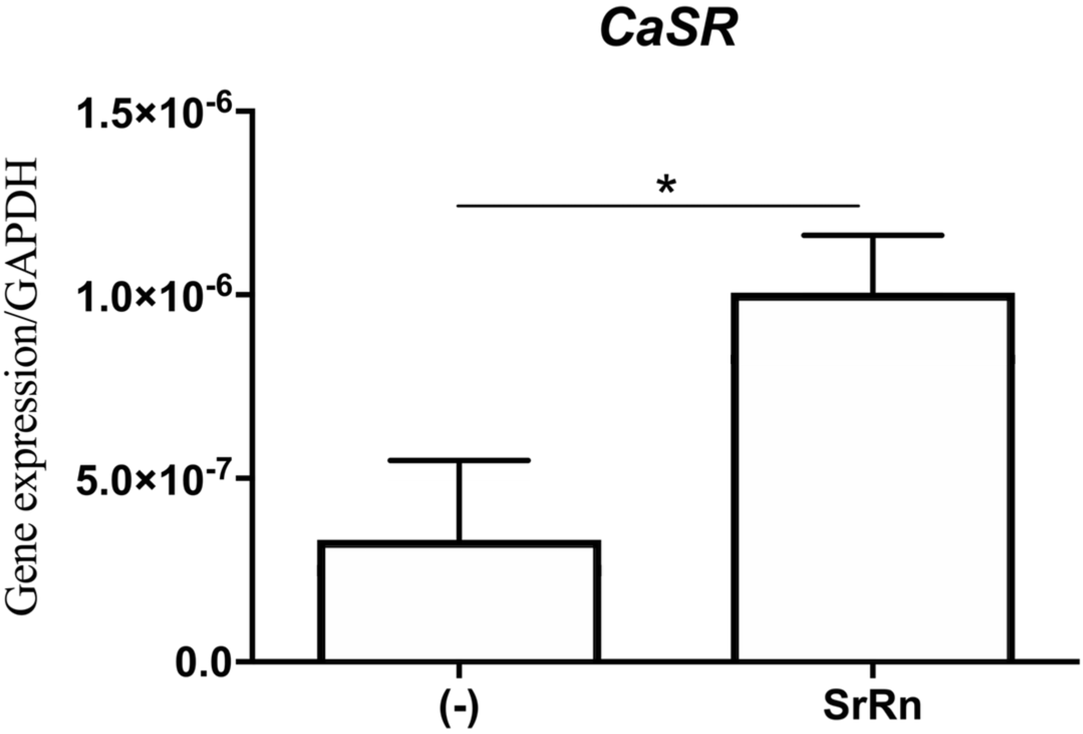
CaSR
1.5×10^-6
1.0×10^-6
5.0×10^-7
0.0
Gene expression/GAPDH
*
(-)
SrRn

Supplemental Figure 1 Effects of $CaCl_2$ on proliferation and mineralized formation in MDPs. (A) WST-8 assay analysis on proliferation of MDPs in the presence or absence of $CaCl_2$ (0.1 or 1.0 mM) at 72 h. (B) $CaCl_2$ did not promote mineralized nodule formation in MDPs.

Supplemental Figure 2 Effects of SrRn on proliferation of human dental pulp cells (hDPCs). Proliferation of hDPCs is promoted by SrRn (1.0 mM) and is blocked by NPS-2143 (1.0 μM) and LY294002 (1.0 μM) at 7 d. *$P<0.05$ or **$p<0.001$ compared with each other.

Supplemental Figure 3 mRNA expression of *CaSR* in MDPs is up-regulated by SrRn. *$P<0.05$ compared with each other.
